# Development of a predictive nomogram for acute respiratory distress syndrome in patients with acute pancreatitis complicated with acute kidney injury

**DOI:** 10.1080/0886022X.2023.2251591

**Published:** 2023-09-19

**Authors:** Dongliang Yang, Jian Kang, Yuanhao Li, Chao Wen, Suosuo Yang, Yanbo Ren, Hui Wang, Yuling Li

**Affiliations:** a Cangzhou Medical College, Cangzhou, P.R. China; b Emergency Department, The First Affiliated Hospital of Dalian Medical University, Dalian, P.R. China; c Department of Anesthesia, The First Affiliated Hospital of Dalian Medical University, Dalian, P.R. China

**Keywords:** Acute pancreatitis, acute respiratory distress syndrome, acute kidney injury, nomogram

## Abstract

**Background:**

Acute respiratory distress syndrome (ARDS) is a common complication in patients with acute pancreatitis (AP), especially when patients complicated with acute kidney injury (AKI), resulting in increased duration of hospitalization and mortality. It is of potential clinical significance to develop a predictive model to identify the the high-risk patients.

**Method:**

AP patients complicated with AKI from January 2019 to March 2022 were enrolled in this study and randomly divided into training cohort and validation cohort at a ratio of 2:1. The Least absolute shrinkage and selection operator(LASSO) regression and machine learning algorithms were applied to select features. A nomogram was developed based on the multivariate logistic regression. The performance of the nomogram was evaluated by AUC, calibration curves, and decision curve analysis.

**Results:**

A total of 292 patients were enrolled in the study, with 206 in the training cohort and 86 in the validation cohort. Multivariate logistic analysis showed that IAP (Odds Ratio (OR)=4.60, 95%CI:1.23-18.24, *p* = 0.02), shock (OR = 12.99, 95%CI:3.47-64.04, *p* < 0.001), CRP(OR= 26.19, 95%CI:9.37-85.57, *p* < 0.001), LDH (OR = 13.13, 95%CI:4.76-40.42, *p* < 0.001) were independent predictors of ARDS. The nomogram was developed based on IAP, shock, CRP and LDH. The nomogram showed good discriminative ability with an AUC value of 0.954 and 0.995 in the training and validation cohort, respectively. The calibration curve indicating good concordance between the predicted and observed values. The DCA showed favorable net clinical benefit.

**Conclusion:**

This study developed a simple model for predicting ARDS in AP patients complicated with AKI. The nomogram can help clinicians identify high-risk patients and optimize therapeutic strategies.

## 1. Introduction

Acute respiratory distress syndrome (ARDS) is one of the most common complications in patients with acute pancreatitis (AP), especially in those complicated with acute kidney injury (AKI) [1]. AKI adversely affect the lungs through kidney-lung crosstalk, and the combination of these two complications significantly worsens outcomes, including higher mortality and increased hospital length-of-stay [2,3].

The pathophysiological mechanism of kidney-lung interactions has been investigated in recent years [4]. Systemic inflammatory response, oxidative stress, production of cytokines/mediators, and leukocyte infiltration are typically activated during AKI, leading to apoptosis of alveolar cells, increased pulmonary vascular permeability, downregulation of pulmonary ion and water transport channels [5]. ARDS, in turn, facilitates and exacerbates AKI *via* metabolic and biomechanical dysregulation.

Fluid management optimization, anti-inflammatory therapy, and blood purification techniques are currently recommended as treatment options for critically ill patients with simultaneous kidney/lung dysfunction [6]. Early identification of the high-risk population for ARDS after AKI in AP patients has potential clinical significance. It can help optimize therapeutic strategies and reduce deleterious kidney-lung crosstalk. However, there exists no model sufficiently reliable to predict ARDS in AP patients complicated with AKI.

Machine learning(ML) is being used in clinical practice for disease prediction or treatment decisions. Among several methods, a random forest (RF) algorithm can handle datasets with many predictive parameters, minimize the potential overfitting of variables and show high practical performance [7]. As a visualization model, nomogram has been widely used in clinical prediction and prognostic research [8].

In this study, we aim to develop a simple and applicable nomogram for predicting ARDS in AP patients complicated with AKI to help clinicians identify high-risk patients and optimize therapeutic strategies.

## 2. Methods

### 2.1. Study design and participants

All AP patients complicated with AKI admitted to the First Affiliated Hospital of Dalian Medical University (Dalian City, Liaoning Province, China) within 72 h of symptom onset from January 2019 to March 2022 were enrolled in this retrospective study. ARDS occurred within 1 week of the onset of AP and followed the diagnosis of AKI. Exclusion criteria were as follows: (1) age under 18 years; (2) malignant tumors; (3) chronic pancreatitis or recurrent pancreatitis; (4) chronic kidney disease; (5) abdominal trauma or surgeries; (6) organ failure caused by underlying diseases (such as heart failure, chronic obstructive pulmonary disease and diabetic ketoacidosis); (7) pregnancy and the immune-deficiency disorders; (8) with incomplete medical data (percent of missing data more than 50%). The enrolled participants was randomly assigned to the training and validation cohort in a 2:1 ratio.

### 2.2. Definitions

According to the guidelines for the management of AP [3,9], acute pancreatitis can be diagnosed based on the fulfillment of two or more of the following: clinical symptoms (upper abdominal pain); laboratory results (the level of serum amylase or lipase is more than three-times the upper limit of the normal value); and/or imaging criteria (CT, MRI, ultrasonography). The severity of AP were evaluated based on the revised Atlanta classification (2012) [10].

AKI was diagnosed based on the 2012 KDIGO guidelines as follows [11]: increase in Scr ≥26.5 μmol/L (0.3 mg/dl) within 48 h, or an increase from the baseline Scr value by ≥50%, or the urine volume less than 0.5 mL/kg/h for >6 h. Baseline SCr is defined as the lowest Scr in the past 7 days.

AKI stage 1 was defined as at least *a* ≥ 50% rise or *a* ≥ 0.3 mg/dl rise in Scr from baseline; AKI stage2 as a 2.0 to 2.9-fold increase in Scr from baseline and AKI stage 3 as a 3.0-fold increase in Scr from baseline or increase in serum creatinine to ≥4.0 mg/dl or initiation of renal replacement therapy.

This study adopted the diagnostic criteria for the New Global Definition of ARDS based on Berlin’s definition [12], combined with changes in clinical practice, especially the use of respiratory support devices [13], which included: (1) ARDS occurred within 1 week of the onset of AP and followed the diagnosis of AKI; (2) Chest imaging (chest radiograph/CT scan) with bilateral opacities not fully explained by effusions, lobar or lung collapse, or nodules; (3) respiratory failure not be fully explained by cardiac failure or fluid overload; (4) For non-intubated patients, PaO_2_/FiO_2_≤300mmHg on high flow nasal oxygen (HFNO) with a flow of 30 L/min or noninvasive ventilation (NIV)/continuous positive airway pressure (CPAP) with at least 5 cmH2O expiratory pressure; 5) For intubated patients, PaO_2_/FiO_2_ ≤300mmHg when a minimum positive end-expiratory pressure (PEEP) of 5 cmH_2_O is required. ARDS is classified as 3 categories: mild ARDS (200 mmHg < PaO_2_/FiO_2_≤300mmHg), moderate ARDS (100 mmHg < PaO_2_/FiO_2_≤200mmHg), and severe ARDS (PaO_2_/FiO_2_≤100 mmHg).

Intra-abdominal hypertension (IAH) is defined by a sustained or repeated pathological elevation in IAP ≥12 mmHg, and abdominal compartment syndrome (ACS) is defined as a sustained IAP > 20 mmHg that is associated with new organ dysfunction/failure caused by AP [14]. Shock was defined as lactate value above 2 mmol/L and persistent hypotension requiring the use of vasopressors to keep mean arterial blood pressure (MAP) above 65 mmHg [15].

### 2.3. Data collection

The medical records of enrolled patients within 48 h after admission were collected from the electronic database of the First Affiliated Hospital of Dalian Medical University, including demographic characteristics, comorbidities information, etiology, laboratory test results, imaging findings and treatment. Scoring systems including Ranson score, Acute Physiology and Chronic Health Evaluation II (APACHE II), computed tomography severity index (CTSI), the bedside index for severity inacute pancreatitis (BISAP) and Sequential organ failure assessment score (SOFA) were evaluated [16].

### 2.4. Statistical analysis

For development and validation of the nomogram, the participants were randomly divided into training cohort and validation cohort at a ratio of 2:1, respectively. Continuous variables were presented as the mean ± standard deviation for parametric variables and as the median (interquartile ranges) for non-parametric variables, and were compared using the student’s t-test or Mann–Whitney *U*-test. Categorical variables were presented as frequencies and proportions, and were compared using Chi-square or Fisher exact test.

Missing data were inputted using multiple imputation methods based on the random forest algorithm, which has shown to be an effective method for imputation [17,18]. The distribution of data did not change after the imputation.

Predictive features were selected using the Least Absolute Shrinkage and Selection Operator (LASSO) regression and random forest (RF) algorithm that can handle high-dimensional and/or multivariate datasets and minimize the potential collinearity and overfitting of variables. Factors associated with ARDS risk in AP patients in the literature were considered as candidate predictors for LASSO and RF algorithms. The common variables were retained for further multivariate logistic regression analysis. Due to the large variance and skewed distribution of the variables, they are transformed into binary variables. Ultimately, independent predictors (*p* < 0.05) assessed by multivariate logistic regression were recruited to develop the nomogram for predicting the occurrence of ARDS.

The discriminative power of the nomogram was assessed by calculating the area under the receiver operating characteristic (ROC) curves (AUC). The calibration curve was applied to evaluate the consistency between the predicted probabilities and the actual outcomes. The clinical utility of the nomograms was measured by decision curve analysis (DCA).

Statistical analysis was performed using SPSS software(version 24.0, Chicago, IL) and R (version 4.1.3) software. The following packages in R were used: glmnet, boruta, rms, pROC, and ggDCA. Two-sided p value < 0.05 was considered to be statistically significant.

## 3. Results

### 3.1. Baseline characteristics

**Figure 1.**
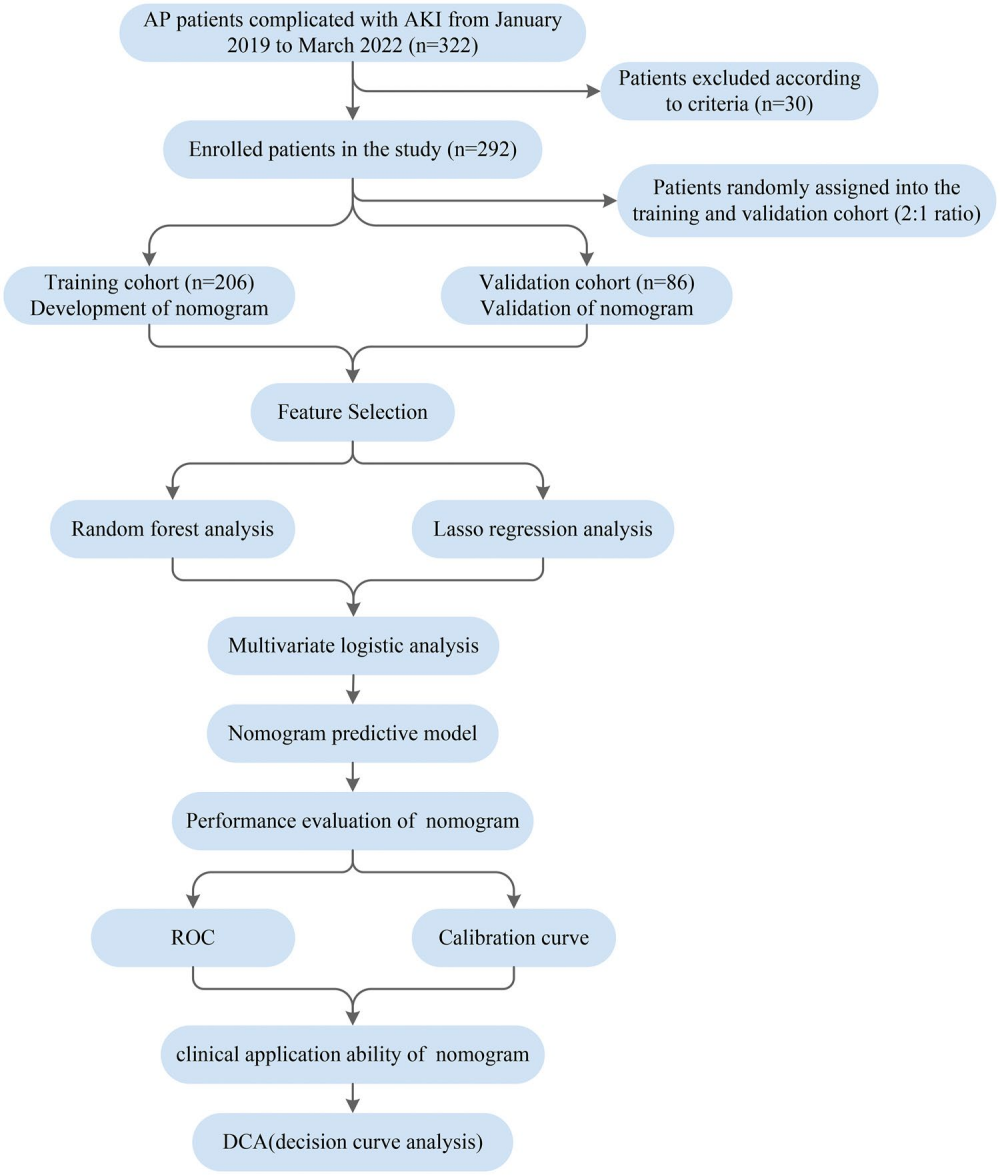
Flow chart of the study.

A total of 322 AP patients complicated with AKI within 48 h after admission to the hospital were initially screened and 30 of those were excluded according to the excluded criteria. ARDS occurred within 1 week of the onset of AP and followed the diagnosis of AKI. Finally, 292 patients were enrolled in the study, including 136 ARDS patients and 156 non-ARDS patients (Figure 1).

The demographic, clinical information and laboratory parameters of all enrolled patients are shown in Table 1. Among the ARDS patients, 49 cases were mild ARDS, 65 cases were moderate ARDS, and 22 cases were severe ARDS (Table 1). The patients were randomly assigned to the training and validation cohort in a 2:1 ratio.

**Table 1.** Baseline characteristics of ARDS and non-ARDS patients in AP patients complicated with AKI.

Variables	Total (*n* = 292)	ARDS (*n* = 136)	N-ARDS (*n* = 156)	*p*
Severity of ARDS, *n* (%)				< 0.001
Mild	49 (17)	49 (36)	0 (0)	
Moderate	65 (22)	65 (48)	0 (0)	
Severe	22 (8)	22 (16)	0 (0)	
N-ARDS	156 (53)	0 (0)	156 (100)	
PaO_2_/FiO_2_, median (Q1,Q3)	310 (189, 338.5)	185 (108, 220.75)	336.5 (323.75, 356)	< 0.001
AKI stage, *n* (%)				< 0.001
Stage 1	143 (49)	45 (33)	98 (63)	
Stage 2	88 (30)	48 (35)	40 (26)	
Stage 3	61 (21)	43 (32)	18 (12)	
Cr, Median (Q1,Q3)	196 (129, 275)	220 (168, 347.25)	156.5 (114, 228.5)	< 0.001
Severity of AP, *n* (%)				< 0.001
MSAP	159 (54)	33 (24)	126 (81)	
SAP	133 (46)	103 (76)	30 (19)	
Sex, *n* (%)				0.511
Female	86 (29)	37 (27)	49 (31)	
Male	206 (71)	99 (73)	107 (69)	
Age, median (Q1,Q3)	62 (45, 75)	56.5 (43, 70)	65.5 (47.75, 79)	0.003
BMI, median (Q1,Q3)	25.9 (22.4, 27.7)	26.7 (22.8, 28.9)	25.5 (22.32, 26.72)	< 0.001
Smoking, *n* (%)				0.055
0	204 (70)	87 (64)	117 (75)	
1	88 (30)	49 (36)	39 (25)	
Alcohol intake, *n* (%)				0.05
0	167 (57)	69 (51)	98 (63)	
1	125 (43)	67 (49)	58 (37)	
HBP, *n* (%)				0.462
0	177 (61)	86 (63)	91 (58)	
1	115 (39)	50 (37)	65 (42)	
DM, *n* (%)				0.065
0	216 (74)	108 (79)	108 (69)	
1	76 (26)	28 (21)	48 (31)	
CHD, *n* (%)				0.122
0	214 (73)	106 (78)	108 (69)	
1	78 (27)	30 (22)	48 (31)	
HF, *n* (%)				< 0.001
0	238 (82)	99 (73)	139 (89)	
1	54 (18)	37 (27)	17 (11)	
Etiology, *n* (%)				0.67
Alchol	37 (13)	19 (14)	18 (12)	
Biliary	127 (43)	54 (40)	73 (47)	
Hypertriglyceridemia	88 (30)	43 (32)	45 (29)	
Others	40 (14)	20 (15)	20 (13)	
Temperature, median (Q1,Q3)	36.7 (36.4, 37.4)	36.8 (36.5, 37.5)	36.6 (36.4, 37.23)	0.286
SBP, median (Q1,Q3)	125 (110, 130.5)	120 (89.75, 130)	130 (120, 135)	< 0.001
DBP, median (Q1,Q3)	60 (50, 74.25)	55 (40, 60)	70 (60, 80)	< 0.001
MAP, median (Q1,Q3)	83 (73, 93)	80 (57, 83)	87 (82, 97.25)	< 0.001
IAP, median (Q1,Q3)	13.5 (11, 17)	17.5 (16, 20)	12 (10, 12)	< 0.001
MODS, *n* (%)				< 0.001
0	77 (26)	5 (4)	72 (46)	
1	215 (74)	131 (96)	84 (54)	
Shock, *n* (%)				< 0.001
0	229 (78)	85 (62)	144 (92)	
1	63 (22)	51 (38)	12 (8)	
Coagulationdysfunction, *n* (%)				0.039
0	197 (67)	83 (61)	114 (73)	
1	95 (33)	53 (39)	42 (27)	
neurological dysfunction, *n* (%)				< 0.001
0	227 (78)	82 (60)	145 (93)	
1	65 (22)	54 (40)	11 (7)	
Gastrointestinal dysfunction, *n* (%)				< 0.001
0	160 (55)	52 (38)	108 (69)	
1	132 (45)	84 (62)	48 (31)	
Operation, *n* (%)				0.058
0	226 (77)	98 (72)	128 (82)	
1	66 (23)	38 (28)	28 (18)	
BISAP, *n* (%)				< 0.001
1	8 (3)	0 (0)	8 (5)	
2	102 (35)	21 (15)	81 (52)	
3	126 (43)	59 (43)	67 (43)	
4	47 (16)	47 (35)	0 (0)	
5	9 (3)	9 (7)	0 (0)	
CTSI, *n* (%)				< 0.001
1	39 (13)	3 (2)	36 (23)	
2	35 (12)	14 (10)	21 (13)	
3	101 (35)	54 (40)	47 (30)	
4	66 (23)	33 (24)	33 (21)	
5	16 (5)	8 (6)	8 (5)	
6	21 (7)	11 (8)	10 (6)	
7	5 (2)	4 (3)	1 (1)	
8	8 (3)	8 (6)	0 (0)	
10	1 (0)	1 (1)	0 (0)	
RANSON, *n* (%)				< 0.001
1	5 (2)	0 (0)	5 (3)	
2	16 (5)	4 (3)	12 (8)	
3	60 (21)	24 (18)	36 (23)	
4	95 (33)	33 (24)	62 (40)	
5	86 (29)	46 (34)	40 (26)	
6	27 (9)	26 (19)	1 (1)	
7	3 (1)	3 (2)	0 (0)	
APACHE-II, median (Q1,Q3)	20 (16, 24.25)	23 (20, 27)	19 (14, 22)	< 0.001
SOFA, median (Q1,Q3)	8.5 (6, 10)	9 (7, 10)	8 (6, 9)	< 0.001
Amy, median (Q1,Q3)	489 (163, 1018)	506 (163.75, 1064)	465.5 (162, 927)	0.476
LPS, median (Q1,Q3)	1361(575.5, 2488)	1373.5 (602.75, 2607)	1361 (568, 2436.5)	0.803
Lac, median (Q1,Q3)	2.6 (1.9, 3.5)	3.55 (2.5, 4.35)	2.1 (1.8, 2.8)	< 0.001
PCT, median (Q1,Q3)	2.73 (0.76, 6.11)	3.25 (0.76, 12.12)	2.5 (0.57, 3.6)	< 0.001
CRP, median (Q1,Q3)	95 (79, 125)	126 (107.75, 148)	82.5 (68.25, 89.25)	< 0.001
WBC, median (Q1,Q3)	12.54 (8.9, 18.67)	13.26 (9.51, 18.8)	12.37 (8.45, 18.54)	0.152
PLT, median (Q1,Q3)	179 (116, 236.25)	183 (111.75, 236.25)	170 (119.75, 236)	0.634
Hb, mean ± SD	126.95 ± 27.62	124.96 ± 26.23	128.69 ± 28.75	0.248
Hct, mean ± SD	38.97 ± 8.52	38.97 ± 8.6	38.96 ± 8.47	0.993
CysC, median (Q1,Q3)	5.21 (4.14, 7.58)	5.51 (4.38, 7.58)	5.21 (4.1, 7.58)	0.342
BUN, median (Q1,Q3)	10.86(7.01, 15.05)	12.02 (8.24, 17.11)	9.5 (6.88, 13.67)	0.003
UA, median (Q1,Q3)	420 (313, 498.25)	456.5 (325.4, 521.25)	401 (300.75, 484)	0.08
LDH, median (Q1,Q3)	169 (125, 202.25)	204.5(186.75, 251.25)	130 (104.5, 160)	< 0.001
CRP/ALB, median (Q1,Q3)	3.62 (2.98, 4.49)	3.74 (3.12, 4.9)	3.48 (2.87, 4.29)	0.003
Cr/ALB, median (Q1,Q3)	6.12 (3.78, 9.17)	7.62 (5.11, 10.39)	4.58 (3.38, 7.49)	< 0.001
Na+, median (Q1,Q3)	138 (134, 141)	137 (133, 140.07)	138 (134, 141)	0.155
Cl-, median (Q1,Q3)	102 (98, 106)	100 (96, 106)	102 (98, 106)	0.049
HCO3-, median (Q1,Q3)	18.7 (15.75, 20.6)	17.4 (13.97, 20)	19 (16.88, 21.58)	< 0.001
BE, median (Q1,Q3)	−7.75(-11.53, −4.18)	−8.65 (-13.43, −4.57)	−7.15 (-10.43, −2.98)	0.012
AG, median (Q1,Q3)	17.05 (13, 22.4)	18.25 (14, 24.02)	15.55 (12.38, 20.52)	< 0.001
K^+^, median (Q1,Q3)	4.24 (3.8, 4.8)	4.42 (3.92, 4.93)	4.12 (3.7, 4.7)	0.005
Ca^2+^, median (Q1,Q3)	1.98 (1.74, 2.15)	1.9 (1.68, 2.1)	2.04 (1.87, 2.18)	< 0.001
Glu, median (Q1,Q3)	8.92 (6.9, 12.11)	9.9 (8.48, 13.25)	7.85 (5.88, 10.51)	< 0.001
PT, median (Q1,Q3)	13.45 (12, 15.43)	13.6 (12.1, 16)	13 (12, 14.83)	0.053
APTT, median (Q1,Q3)	29.45(24.87, 35.82)	30.75 (25.17, 37.08)	28.65 (24.58, 34.68)	0.072
D-Dimer, median (Q1,Q3)	2290 (1225, 4449)	2785 (1278.5, 5910)	2100 (1070, 3835)	0.004
CK-MB, median (Q1,Q3)	1.9 (0.65, 3.96)	2.39 (0.63, 5.54)	1.51 (0.65, 3.36)	0.079
hs-cTnI, median (Q1,Q3)	0.04 (0.01, 0.13)	0.07 (0.02, 0.19)	0.03 (0.01, 0.08)	< 0.001
BNP, Median (Q1,Q3)	118.5 (70.75, 260)	124 (67.5, 349.25)	108 (75.75, 225)	0.453
ALB, Mean ± SD	32.8 ± 5.99	32.21 ± 6.03	33.32 ± 5.93	0.113
T-BIL, median (Q1,Q3)	21.35 (13.2, 35.75)	21.8 (14.67, 35.95)	20 (12.85, 35)	0.344
D-BIL, median (Q1,Q3)	7.1 (3.35, 13.7)	8.65 (3.58, 14.62)	6.55 (3.35, 13)	0.313
ALT, Median (Q1,Q3)	44.5 (22, 100.25)	44.5 (22, 88.5)	45.5 (21.3, 115.5)	0.87
AST, Median (Q1,Q3)	57.5 (31, 145.25)	68.5 (34.92, 174.25)	54 (30, 137)	0.108
ALP, Median (Q1,Q3)	85.5 (66, 128.25)	80 (63, 115.25)	89 (67, 136)	0.046
Γ-GGT, median (Q1,Q3)	84.5 (39.75, 210)	78.5 (37.75, 198.5)	85 (41.75, 216.25)	0.769
NH3, Median (Q1,Q3)	25 (16, 31)	25 (16.75, 36)	25 (16, 30)	0.107
LDL-C, median (Q1,Q3)	2.9 (1.98, 4.09)	2.89 (1.92, 3.69)	2.94 (2.05, 4.5)	0.134
HDL-C, median (Q1,Q3)	0.9 (0.6, 1.4)	0.8 (0.6, 1.22)	0.92 (0.66, 1.46)	0.081
TG, Median (Q1,Q3)	3.72 (2.31, 5.85)	4.55 (3.16, 7.59)	3.06 (1.96, 5.38)	< 0.001
TC, Median (Q1,Q3)	3.87 (3.2, 5.01)	4.12 (3.3, 5.64)	3.66 (3.1, 4.66)	0.003
Ventilation, *n* (%)				< 0.001
HFNC	97 (33)	67 (49)	30(19)	
IMV	26 (9)	20 (15)	6(4)	
NIV	22 (8)	16 (12)	6(4)	
Oxygen	147 (50)	33 (24)	114(73)	
CRRT, *n* (%)				< 0.001
0	233 (80)	96 (71)	137(88)	
1	59 (20)	40 (29)	19(12)	

MSAP: moderately severe acute pancreatitis; SAP: severe acute pancreatitis; AKI: acute kidney injury; BMI: body mass index; SBP: systolic blood pressure; DBP: diastolic blood pressure; MAP: mean arterial pressure; MODS: multiple organ dysfunction syndrome; ARDS: acute respiratory distress syndrome; BISAP: bedside index for severity of acute pancreatitis; CTSI: computed tomography severity index; APACHEII: acute physiology and chronic health evaluation II; SOFA: sequential organ failure assessment; Amy: amylase; LPS: lipase; Lac: lactic acid; PCT: procalcitonin; CRP: C-reactive protein; WBC: white blood cell count; PLT: Platelets; Hb: hemoglobin; Hct : hematocrit; CysC: cystatin C; BUN: blood urea nitrogen; UA: Uric Acid; LDH: lactate dehydrogenase; Na^+^: sodium; Cl^-^: chlorine; HCO_3_^-^: bicarbonate; BE: bases excess; AG: anion gap; K^+^: Potassium; Ca^2+^: calcium; Glu: glucose; PT: prothrombin time; APTT: activated partial thromboplastin time; CK-MB: creatine kinase isoenzyme; hs-cTnI: Cardiac troponin I; BNP: B-type natriuretic peptide; ALB: albumin; T-BIL: total bilirubin; D-BIL: direct bilirubin; ALT: alanine amino-transferase; AST: aspartate amino-transferase; ALP: alkaline phosphatase; γ-GGT : γ-glutamyl transpeptadase; NH3: ammonia; LDL-C: Low-density lipoprotein cholesterol; HDL-C: High-density lipoprotein cholesterol; TG: triglyceride; TC: total cholesterol; IQR: inter-quartile range; HFNC: high flow nasal cannula; IMV: Invasive Mechanical Ventilation; NIV: noninvasive ventilation.

The training cohort consisted of 206 patients (96 ARDS cases, 110 non-ARDS cases), and the validation cohort consisted of 86 patients (40 ARDS cases, 46 non-ARDS cases). The incidence of ARDS was comparable between the training and validation cohorts. The baseline characteristics of patients in the training and validation cohorts are listed in Table 2 and 3, respectively.

**Table 2.** Baseline characteristics of ARDS and non-ARDS patients in the training cohort.

Variables	Total (*n* = 206)	ARDS (*n* = 96)	N-ARDS (*n* = 110)	*p*
AKI stage, *n* (%)				< 0.001
Stage 1	105 (51)	34 (35)	71 (65)	
Stage 2	54 (26)	30 (31)	24 (22)	
Stage 3	47 (23)	32 (33)	15 (14)	
Cr, Median (Q1,Q3)(µg/L)	188 (124.5, 279.5)	217.5 (163, 352.75)	155.5 (111.25, 229.5)	< 0.001
Severity of AP, *n* (%)				< 0.001
MSAP	109 (53)	23 (24)	86 (78)	
SAP	97 (47)	73 (76)	24 (22)	
Sex, *n* (%)				1
female	59 (29)	27 (28)	32 (29)	
male	147 (71)	69 (72)	78 (71)	
Age, median (Q1,Q3)(year)	61 (45, 74.75)	55 (43, 67.25)	66.5 (47.25, 79.75)	0.001
BMI, median(Q1,Q3)(kg/m²)	25.8 (22.15, 27.6)	26.6 (22.37, 28.5)	25.5 (21.92, 26.78)	0.025
Smoking, *n* (%)				0.084
0	138 (67)	58 (60)	80 (73)	
1	68 (33)	38 (40)	30 (27)	
Alcohol intake, *n* (%)				0.121
0	118 (57)	49 (51)	69 (63)	
1	88 (43)	47 (49)	41 (37)	
Hypertension, *n* (%)				0.225
0	125 (61)	63 (66)	62 (56)	
1	81 (39)	33 (34)	48 (44)	
Diabetes, n (%)				0.307
0	153 (74)	75 (78)	78 (71)	
1	53 (26)	21 (22)	32 (29)	
Coronary heart disease, *n* (%)				0.29
0	155 (75)	76 (79)	79 (72)	
1	51 (25)	20 (21)	31 (28)	
Heart failure, *n* (%)				0.005
0	170 (83)	71 (74)	99 (90)	
1	36 (17)	25 (26)	11 (10)	
Etiology, *n* (%)				0.432
Alchol	29 (14)	16 (17)	13 (12)	
Biliary	87 (42)	35 (36)	52 (47)	
Hypertriglyceridemia	63 (31)	31 (32)	32 (29)	
Others	27 (13)	14 (15)	13 (12)	
Temperature,Median (Q1,Q3)(°C)	36.7 (36.4, 37.4)	36.8 (36.5, 37.5)	36.6 (36.4, 37.4)	0.515
SBP, Median (Q1,Q3)(mmHg)	125 (110, 130)	120 (88.75, 130)	128 (120, 135)	< 0.001
DBP, Median (Q1,Q3)(mmHg)	60 (50, 70.75)	55 (40, 60)	70 (60, 80)	< 0.001
MAP, Median (Q1,Q3)(mmHg)	83 (72.25, 93)	78.5 (57, 83)	87 (82.25, 97)	< 0.001
IAP, Median (Q1,Q3)(mmHg)	12 (11, 17)	17.5 (15, 20)	12 (10, 12)	< 0.001
MODS, *n* (%)				< 0.001
0	50 (24)	4 (4)	46 (42)	
1	156 (76)	92 (96)	64 (58)	
Shock, *n* (%)				< 0.001
0	160 (78)	58 (60)	102 (93)	
1	46 (22)	38 (40)	8 (7)	
Coagulation dysfunction, *n* (%)				0.404
0	138 (67)	61 (64)	77 (70)	
1	68 (33)	35 (36)	33 (30)	
Neurological dysfunction, *n* (%)				< 0.001
0	158 (77)	58 (60)	100 (91)	
1	48 (23)	38 (40)	10 (9)	
Gastrointestinal dysfunction, *n* (%)				< 0.001
0	106 (51)	34 (35)	72 (65)	
1	100 (49)	62 (65)	38 (35)	
Operation, *n* (%)				0.126
0	157 (76)	68 (71)	89 (81)	
1	49 (24)	28 (29)	21 (19)	
BISAP, *n* (%)				< 0.001
1	5 (2)	0 (0)	5 (5)	
2	73 (35)	16 (17)	57 (52)	
3	88 (43)	40 (42)	48 (44)	
4	34 (17)	34 (35)	0 (0)	
5	6 (3)	6 (6)	0 (0)	
CTSI, *n* (%)				< 0.001
1	31 (15)	3 (3)	28 (25)	
2	25 (12)	11 (11)	14 (13)	
3	62 (30)	32 (33)	30 (27)	
4	50 (24)	27 (28)	23 (21)	
5	11 (5)	4 (4)	7 (6)	
6	15 (7)	8 (8)	7 (6)	
7	4 (2)	3 (3)	1 (1)	
8	8 (4)	8 (8)	0 (0)	
10	0(0)	0 (0)	0 (0)	
RANSON, *n* (%)				< 0.001
1	4 (2)	0 (0)	4 (4)	
2	12 (6)	2 (2)	10 (9)	
3	45 (22)	18 (19)	27 (25)	
4	62 (30)	22 (23)	40 (36)	
5	61 (30)	33 (34)	28 (25)	
6	20 (10)	19 (20)	1 (1)	
7	2 (1)	2 (2)	0 (0)	
APACHE II, Median (Q1,Q3)	20 (16, 24)	23.5 (19, 27)	19 (14.25, 22)	< 0.001
SOFA, Median (Q1,Q3)	9 (6, 10)	9 (7, 10)	8 (6, 9)	< 0.001
Amy, Median (Q1,Q3)(U/L)	480 (171.5, 1056.25)	603 (234.25, 1064)	424.85(140.25, 1056.25)	0.222
LPS, Median (Q1,Q3)(U/L)	1369 (612.75, 2581.75)	1373.5(727.5, 2609.75)	1369 (568, 2581.75)	0.921
Lac, Median (Q1,Q3)(mmol/L)	2.7 (1.8, 3.58)	3.55 (2.5, 4.6)	2.2 (1.8, 2.8)	< 0.001
PCT, Median (Q1,Q3)(ng/L)	2.8 (0.8, 6.07)	3.56 (0.9, 10.64)	2.43 (0.5, 3.76)	0.001
CRP, Median (Q1,Q3)(mg/l)	95 (78, 126)	126 (107.75, 148.5)	79 (62.75, 89.75)	< 0.001
WBC, Median (Q1,Q3)(10^9^/L)	12.54 (9.17, 17.91)	14.33 (10.27, 18.81)	11.68 (8.52, 17.42)	0.024
PLT, Median (Q1,Q3) (10^9^/L)	181 (119.25, 236)	202.5 (111.75, 233.75)	171 (121, 239.75)	0.884
Hb, Mean ± SD(g/L)	128.01 ± 28.79	127.55 ± 27.79	128.42 ± 29.76	0.829
Hct, Mean ± SD(%)	39.09 ± 8.89	39.6 ± 9.09	38.64 ± 8.73	0.447
CysC, Median (Q1,Q3)(mg/L)	5.51 (4.32, 7.58)	5.56 (4.49, 7.61)	5.23 (4.15, 7.47)	0.255
BUN, Median (Q1,Q3)(mmol/L)	11.05 (6.9, 15.43)	12.4 (8.25, 17.42)	9.48 (6.73, 13.39)	0.003
UA, Median (Q1,Q3) (umol/L)	420 (305, 502.75)	458.5 (325.85, 530.75)	389.5 (295.25, 481.5)	0.023
LDH, Median (Q1,Q3)(u/L)	174 (125, 205)	206.5 (187.75, 256)	128.5 (102, 160.75)	< 0.001
CRP/ALB, Median (Q1,Q3)	3.63 (2.97, 4.5)	3.82 (3.1, 4.9)	3.42 (2.87, 4.3)	0.015
Cr/ALB, Median (Q1,Q3)	5.84 (3.64, 9.4)	7.72 (5.1, 10.35)	4.57 (3.38, 7.46)	< 0.001
Na^+^, Median (Q1,Q3) (mmol/L)	138 (134, 141)	137 (133.8, 140.48)	138 (134, 141)	0.219
Cl^-^, Median (Q1,Q3) (mmol/L)	102 (98, 106)	100.5 (96.75, 106)	103 (98, 106)	0.215
HCO3^-^,Median(Q1,Q3)(mmol/L)	18.7 (15.8, 20.45)	17.6 (13.95, 20)	18.95 (16.8, 21.3)	0.005
BE, Median (Q1,Q3) (mmol/L)	−8 (-11.6, −4.3)	−9.1 (-12.98, −4.95)	−7.4 (-10.5, −2.95)	0.052
AG, Median (Q1,Q3) (mmol/L)	16.3 (13, 22)	17.7 (13.95, 23.45)	15.1 (12.4, 19.7)	0.008
K^+^, Median (Q1,Q3) (mmol/L)	4.2 (3.8, 4.78)	4.41 (3.9, 4.9)	4.11 (3.7, 4.66)	0.012
Ca^2+^, Median (Q1,Q3) (mmol/L)	1.97 (1.72, 2.16)	1.9 (1.66, 2.06)	2.05 (1.81, 2.18)	0.002
Glu, Median (Q1,Q3) (mmol/L)	8.94 (6.88, 12.04)	10.04 (8.54, 13.05)	7.92 (6.05, 10.18)	< 0.001
PT, Median (Q1,Q3)(s)	13.6 (12.2, 15.47)	13.75 (12.17, 16)	13.45 (12.2, 14.88)	0.208
APTT, Median (Q1,Q3) (s)	29.85 (25.02, 35.18)	31.15 (25.08, 36.28)	28.7 (25.02, 35)	0.451
D-Dimer, Median (Q1,Q3)(mg/L)	2410 (1215, 4815)	3150 (1440, 7637.5)	2100 (1055, 3822.5)	0.001
CK-MB, Median (Q1,Q3)(µg/L)	1.7 (0.65, 3.64)	2.26 (0.9, 5.19)	1.23 (0.64, 3.18)	0.008
hs-cTnI, Median (Q1,Q3) (µg/L)	0.05 (0.02, 0.13)	0.08 (0.02, 0.22)	0.03 (0.01, 0.07)	< 0.001
BNP, Median (Q1,Q3)(pg/ml)	110.5 (68.25, 260)	118.5 (57.5, 325)	103 (75.25, 230)	0.803
ALB, Median (Q1,Q3)(g/L)	32.8 ± 5.97	32.14 ± 5.89	33.38 ± 6.01	0.139
T-BIL, Median (Q1,Q3)(µmol/L)	21.75 (14.45, 35.67)	21.75 (15.33, 35.23)	21.8 (13, 35.62)	0.519
D-BIL, Median (Q1,Q3) (µmol/L)	7.25 (3.2, 13.6)	8.75 (3.85, 14.62)	6.3 (3.1, 12.55)	0.184
ALT, Median (Q1,Q3)(U/L)	42 (22, 97.25)	44 (25, 80.38)	41 (21, 114.5)	0.901
AST, Median (Q1,Q3) (U/L)	57 (30.25, 145)	69 (35.75, 178)	51 (30, 124.75)	0.048
ALP, Median (Q1,Q3) (U/L)	86 (66, 127.5)	77.5 (62.75, 109.5)	93 (68.5, 141.75)	0.016
Γ-GGT, Median (Q1,Q3) (U/L)	84.5 (38.25, 227.5)	71 (34.92, 226.75)	85.5 (45, 227.5)	0.444
NH3, Median (Q1,Q3)(umol/L)	25 (17, 31)	25 (17, 36.5)	22 (16, 30)	0.019
LDL-C, Median (Q1,Q3)(mmol/L)	2.9 (2.05, 4.14)	2.89 (1.97, 3.7)	2.94 (2.14, 4.56)	0.136
HDL-C, Median (Q1,Q3)(mmol/L)	0.89 (0.6, 1.4)	0.8 (0.54, 1.16)	0.96 (0.69, 1.44)	0.037
TG, Median (Q1,Q3) (mmol/L)	3.76 (2.28, 5.74)	4.45 (2.97, 7.59)	3.45 (1.99, 5.4)	0.01
TC, Median (Q1,Q3) (mmol/L)	3.88 (3.19, 4.85)	3.98 (3.25, 5.54)	3.69 (3.13, 4.78)	0.084
Ventilation, n (%)				< 0.001
HFNC	72(35)	51 (53)	21(18)	
IMV	17 (8)	14 (15)	3(3)	
NIV	12 (6)	8 (8)	4(4)	
Oxygen	105 (51)	23 (24)	82(75)	
CRRT, *n* (%)				0.001
0	164 (80)	67 (70)	97(89)	
1	42 (20)	29 (30)	13(11)	

MSAP: moderately severe acute pancreatitis; SAP: severe acute pancreatitis; AKI: acute kidney injury; BMI: body mass index; SBP:systolic blood pressure; DBP:diastolic blood pressure; MAP: mean arterial pressure; MODS: multiple organ dysfunction syndrome; ARDS: acute respiratory distress syndrome; BISAP:bedside index for severity of acute pancreatitis; CTSI:computed tomography severity index; APACHEII:acute physiology and chronic health evaluation II; SOFA: sequential organ failure assessment; Amy: amylase; LPS:lipase; Lac:lactic acid; PCT: procalcitonin; CRP: C-reactive protein; WBC: white blood cell count; PLT:Platelets; Hb: hemoglobin; Hct :hematocrit; CysC:cystatin C; BUN:blood urea nitrogen; UA:Uric Acid; LDH:lactate dehydrogenase; Na^+^: sodium; Cl^-^: chlorine; HCO_3_^-^: bicarbonate; BE:bases excess; AG: anion gap; K^+^: Potassium; Ca^2+^:calcium; Glu:glucose; PT:prothrombin time; APTT:activated partial thromboplastin time; CK-MB: creatine kinase isoenzyme; hs-cTnI: Cardiac troponin I; BNP:B-type natriuretic peptide; ALB:albumin; T-BIL:total bilirubin; D-BIL:direct bilirubin; ALT:alanine amino-transferase; AST:aspartate amino-transferase; ALP:alkaline phosphatase; γ-GGT : γ-glutamyl transpeptadase; NH3: ammonia; LDL-C:Low-density lipoprotein cholesterol; HDL-C:High-density lipoprotein cholesterol; TG:triglyceride; TC:total cholesterol; IQR:inter-quartile range; HFNC:high flow nasal cannula; IMV: Invasive Mechanical Ventilation; NIV: noninvasive ventilation.

**Table 3.** Baseline characteristics of ARDS and non-ARDS patients in the validation cohort.

Variables	Total (*n* = 86)	ARDS (*n* = 40)	N-ARDS (*n* = 46)	*p*
AKI stage, *n* (%)				0.004
Stage 1	38 (44)	11 (28)	27 (59)	
Stage 2	34 (40)	18 (45)	16 (35)	
Stage 3	14 (16)	11 (28)	3 (7)	
Cr, Median (Q1,Q3)(µg/L)	206.5 (136, 260.75)	223 (175.75, 338)	164 (126.75, 227)	0.003
Severity of AP, *n* (%)				< 0.001
MSAP	50 (58)	10 (25)	40 (87)	
SAP	36 (42)	30 (75)	6 (13)	
Sex, n (%)				0.338
female	27 (31)	10 (25)	17 (37)	
male	59 (69)	30 (75)	29 (63)	
Age, Median (Q1,Q3)(year)	61.19 ± 18.48	60.25 ± 18.54	62 ± 18.59	0.664
BMI, Median(Q1,Q3)(kg/m²)	26.1 (23.25, 28.7)	27.6 (25.4, 29.1)	25.7 (22.85, 26.55)	0.006
Smoking, *n* (%)				0.54
0	66 (77)	29 (72)	37 (80)	
1	20 (23)	11 (28)	9 (20)	
Alcohol intake, *n* (%)				0.317
0	49 (57)	20 (50)	29 (63)	
1	37 (43)	20 (50)	17 (37)	
Hypertension, *n* (%)				0.762
0	52 (60)	23 (57)	29 (63)	
1	34 (40)	17 (42)	17 (37)	
Diabetes, *n* (%)				0.118
0	63 (73)	33 (82)	30 (65)	
1	23 (27)	7 (18)	16 (35)	
Coronary heart disease, *n* (%)				0.338
0	59 (69)	30 (75)	29 (63)	
1	27 (31)	10 (25)	17 (37)	
Heart failure, *n* (%)				0.096
0	68 (79)	28 (70)	40 (87)	
1	18 (21)	12 (30)	6 (13)	
Etiology, *n* (%)				0.979
Alchol	8 (9)	3 (8)	5 (11)	
Biliary	40 (47)	19 (48)	21 (46)	
Hypertriglyceridemia	25 (29)	12 (30)	13 (28)	
Others	13 (15)	6 (15)	7 (15)	
Temperature, Median (Q1,Q3)(°C)	36.65 (36.4, 37.27)	36.75 (36.48, 37.42)	36.6 (36.32, 37.2)	0.359
SBP, median (Q1,Q3)(mmHg)	125.5 (120, 131.5)	120 (90, 130)	130 (121.25, 133.75)	0.007
DBP, median (Q1,Q3)(mmHg)	60 (51.25, 79)	60 (46.5, 60)	65.5 (60, 80)	< 0.001
MAP, median (Q1,Q3)(mmHg)	83 (77, 93.75)	80 (59.5, 83)	87 (82.25, 98.75)	< 0.001
IAP, median (Q1,Q3)(mmHg)	14 (11, 17)	17.5 (16, 20)	11.5 (10, 12)	< 0.001
MODS, *n* (%)				< 0.001
0	27 (31)	1 (2)	26 (57)	
1	59 (69)	39 (98)	20 (43)	
Shock, *n* (%)				0.013
0	69 (80)	27 (68)	42 (91)	
1	17 (20)	13 (32)	4 (9)	
Coagulation dysfunction, *n* (%)				0.021
0	59 (69)	22 (55)	37 (80)	
1	27 (31)	18 (45)	9 (20)	
Neurological dysfunction, *n* (%)				< 0.001
0	69 (80)	24 (60)	45 (98)	
1	17 (20)	16 (40)	1 (2)	
Gastrointestinal dysfunction, *n* (%)				0.003
0	54 (63)	18 (45)	36 (78)	
1	32 (37)	22 (55)	10 (22)	
Operation, *n* (%)				0.387
0	69 (80)	30 (75)	39 (85)	
1	17 (20)	10 (25)	7 (15)	
BISAP, *n* (%)				< 0.001
1	3 (3)	0 (0)	3 (7)	
2	29 (34)	5 (12)	24 (52)	
3	38 (44)	19 (48)	19 (41)	
4	13 (15)	13 (32)	0 (0)	
5	3 (3)	3 (8)	0 (0)	
CTSI, *n* (%)				0.015
1	8 (9)	0 (0)	8 (17)	
2	10 (12)	3 (8)	7 (15)	
3	39 (45)	22 (55)	17 (37)	
4	16 (19)	6 (15)	10 (22)	
5	5 (6)	4 (10)	1 (2)	
6	6 (7)	3 (8)	3 (7)	
7	1 (1)	1 (2)	0 (0)	
8	0(0)	0 (0)	0 (0)	
10	1 (1)	1 (2)	0 (0)	
RANSON, *n* (%)				0.018
1	1 (1)	0 (0)	1 (2)	
2	4 (5)	2 (5)	2 (4)	
3	15 (17)	6 (15)	9 (20)	
4	33 (38)	11 (28)	22 (48)	
5	25 (29)	13 (32)	12 (26)	
6	7 (8)	7 (18)	0 (0)	
7	1 (1)	1 (2)	0 (0)	
APACHE II, Median (Q1,Q3)	22 (16, 25)	23 (21.75, 28.5)	19 (13.25, 24)	< 0.001
SOFA, Median (Q1,Q3)	8 (6, 10)	10 (8, 10)	8 (6, 9)	0.002
Amy, Median (Q1,Q3)(U/L)	502.5 (163, 909.5)	372.5 (131, 1068.5)	546 (225.5, 821)	0.53
LPS, Median (Q1,Q3)(U/L)	1361 (463.25, 2388.5)	1443.5 (424.5, 2565)	1361 (656.75, 1986.75)	0.719
Lac, Median (Q1,Q3)(mmol/L)	2.6 (1.9, 3.48)	3.55 (2.55, 3.97)	2.1 (1.9, 2.6)	< 0.001
PCT, Median (Q1,Q3)(ng/L)	2.6 (0.63, 6.05)	3.01 (0.57, 14.11)	2.5 (1.21, 3.5)	0.138
CRP, Median (Q1,Q3)(mg/l)	96.5 (83.25, 119.75)	125 (108.5, 148)	84.5 (71.75, 88.75)	< 0.001
WBC, Median (Q1,Q3)(10^9^/L)	12.17 (7.88, 18.85)	11.59 (7.7, 17.25)	12.69 (8.26, 18.93)	0.505
PLT, Median (Q1,Q3) (10^9^/L)	172 (112, 252)	179 (111.5, 259.5)	169 (119, 221)	0.631
Hb, Mean ± SD(g/L)	124.41 ± 24.58	118.75 ± 21.09	129.33 ± 26.5	0.043
Hct, Mean ± SD(%)	38.67 ± 7.6	37.47 ± 7.19	39.72 ± 7.87	0.17
CysC, Median (Q1,Q3)(mg/L)	4.84 (4.1, 7.58)	4.85 (4.26, 7.54)	4.77 (4.1, 8.31)	0.89
BUN, Median (Q1,Q3)(mmol/L)	10.78 (7.5, 14.29)	11.05 (8.22, 14.73)	9.52 (7.1, 13.92)	0.384
UA, Median (Q1,Q3) (µmol/L)	400.62 ± 143.05	397.21 ± 162.3	403.59 ± 125.72	0.841
LDH, Median (Q1,Q3)(µ/L)	168.5 (128.75, 196)	198.5 (184.5, 241.25)	131.5 (117, 157.5)	< 0.001
CRP/ALB, Median (Q1,Q3)	3.57 (3.07, 4.49)	3.64 (3.26, 4.89)	3.52 (2.89, 4.2)	0.122
Cr/ALB, Median (Q1,Q3)	6.47 (4.13, 8.74)	7.5 (5.37, 10.48)	5 (3.43, 7.47)	0.003
Na^+^, Median (Q1,Q3) (mmol/L)	137 (133.25, 141)	137 (133, 140)	138 (135, 141)	0.524
Cl^-^, Median (Q1,Q3) (mmol/L)	101 (97, 105)	99.5 (96, 103.25)	102 (98, 106)	0.084
HCO_3_^-^,Median(Q1,Q3)(mmol/L)	18.7 (15.65, 21.17)	16.75 (14.05, 20.3)	19.3 (17.55, 22.12)	0.033
BE, Median (Q1,Q3) (mmol/L)	−7.2 (-10.9, −3.72)	−8.3 (-13.45, −4.4)	−7 (-10.1, −3.05)	0.108
AG, Median (Q1,Q3) (mmol/L)	18.95 ± 7.61	21.21 ± 8.19	16.98 ± 6.54	0.011
K^+^, Median (Q1,Q3) (mmol/L)	4.46 ± 0.92	4.57 ± 0.97	4.37 ± 0.87	0.336
Ca^2+^, Median (Q1,Q3) (mmol/L)	1.99 ± 0.31	1.93 ± 0.35	2.04 ± 0.25	0.119
Glu, Median (Q1,Q3) (mmol/L)	8.88 (6.92, 12.94)	9.07 (7.83, 14.05)	7.57 (5.77, 11.81)	0.049
PT, Median (Q1,Q3)(s)	12.7 (11.72, 15.02)	13.45 (12, 15.75)	12.5 (11.43, 14.3)	0.083
APTT, Median (Q1,Q3) (s)	29.4 (24.55, 36.3)	30.35 (26.2, 44.35)	27.4 (23.77, 31.8)	0.049
D-Dimer,Median (Q1,Q3)(mg/L)	1854.5 (1235, 3558.75)	1819 (1245, 3526.25)	2040 (1212.5, 3712.5)	0.866
CK-MB, Median (Q1,Q3)(ug/L)	2.34 (0.61, 5.97)	2.68 (0.47, 6.23)	2.26 (1.03, 4.82)	0.588
hs-cTnI, Median (Q1,Q3) (µg/L)	0.04 (0.01, 0.12)	0.04 (0.01, 0.17)	0.03 (0.01, 0.11)	0.544
BNP, Median (Q1,Q3)(pg/ml)	155.5 (86.25, 259.5)	191 (86.75, 650)	129.5 (86.75, 220)	0.347
ALB, Median (Q1,Q3)(g/L)	32.81 ± 6.06	32.36 ± 6.41	33.19 ± 5.78	0.533
T-BIL, Median (Q1,Q3)(µmol/L)	19.9 (12.9, 35.53)	22.45 (12.97, 37.03)	18.85 (12.75, 33.38)	0.533
D-BIL, Median (Q1,Q3) (µmol/L)	6.9 (3.52, 15.72)	6.7 (3.13, 13.4)	7.2 (3.62, 16.03)	0.775
ALT, Median (Q1,Q3)(U/L)	52 (22, 120.25)	45.5 (20.5, 126.75)	56 (24.25, 114)	0.659
AST, Median (Q1,Q3) (U/L)	61.5 (33, 160.5)	65.5 (33, 130.85)	59.5 (33.5, 170.75)	0.897
ALP, Median (Q1,Q3) (U/L)	83.5 (66, 128.5)	83.5 (65, 125.5)	84.5 (67, 129.25)	0.955
Γ-GGT, Median (Q1,Q3) (U/L)	84.5 (41.25, 173.75)	108.95 (44.25, 167.5)	78 (39.5, 173.75)	0.497
NH3, Median (Q1,Q3)(µmol/L)	25 (15, 30.75)	21 (13, 33.75)	28 (18, 30)	0.453
LDL-C,Median (Q1,Q3)(mmol/L)	2.94 (1.83, 3.79)	2.92 (1.76, 3.64)	2.94 (1.97, 4.1)	0.594
HDL-C, Median (Q1,Q3)(mmol/L)	0.9 (0.64, 1.39)	0.9 (0.7, 1.31)	0.88 (0.61, 1.48)	0.924
TG, Median (Q1,Q3) (mmol/L)	3.61 (2.42, 6.8)	4.78 (3.31, 7.55)	2.7 (1.91, 4.86)	<0.001
TC, Median (Q1,Q3) (mmol/L)	3.84 (3.22, 5.2)	4.48 (3.68, 5.78)	3.56 (3.08, 4.2)	0.009
Ventilation, *n* (%)				<0.001
HFNC	26 (30)	16 (40)	10(21)	
IMV	9 (10)	6 (15)	3(6)	
NIV	10 (11)	8 (20)	2(4)	
Oxygen	41 (48)	10 (25)	31(68)	
CRRT, *n* (%)				0.238
0	68 (79)	29 (72)	39(85)	
1	18 (21)	11 (28)	7(15)	

MSAP: moderately severe acute pancreatitis; SAP: severe acute pancreatitis; AKI: acute kidney injury; BMI: body mass index; SBP:systolic blood pressure; DBP:diastolic blood pressure; MAP: mean arterial pressure; MODS: multiple organ dysfunction syndrome; ARDS: acute respiratory distress syndrome; BISAP:bedside index for severity of acute pancreatitis; CTSI:computed tomography severity index; APACHEII:acute physiology and chronic health evaluation II; SOFA: sequential organ failure assessment; Amy: amylase; LPS:lipase; Lac:lactic acid; PCT: procalcitonin; CRP: C-reactive protein; WBC: white blood cell count; PLT:Platelets; Hb: hemoglobin; Hct :hematocrit; CysC:cystatin C; BUN:blood urea nitrogen; UA:Uric Acid; LDH:lactate dehydrogenase; Na^+^: sodium; Cl^-^: chlorine; HCO_3_^-^: bicarbonate; BE:bases excess; AG: anion gap; K^+^: Potassium; Ca^2+^:calcium; Glu:glucose; PT:prothrombin time; APTT:activated partial thromboplastin time; CK-MB: creatine kinase isoenzyme; hs-cTnI: Cardiac troponin I; BNP:B-type natriuretic peptide; ALB:albumin; T-BIL:total bilirubin; D-BIL:direct bilirubin; ALT:alanine amino-transferase; AST:aspartate amino-transferase; ALP:alkaline phosphatase; γ-GGT : γ-glutamyl transpeptadase; NH3: ammonia; LDL-C:Low-density lipoprotein cholesterol; HDL-C:High-density lipoprotein cholesterol; TG:triglyceride; TC:total cholesterol; IQR:inter-quartile range; HFNC:high flow nasal cannula; IMV: Invasive Mechanical Ventilation; NIV: noninvasive ventilation.

### 3.2. Feature selection

**Figure 2.**
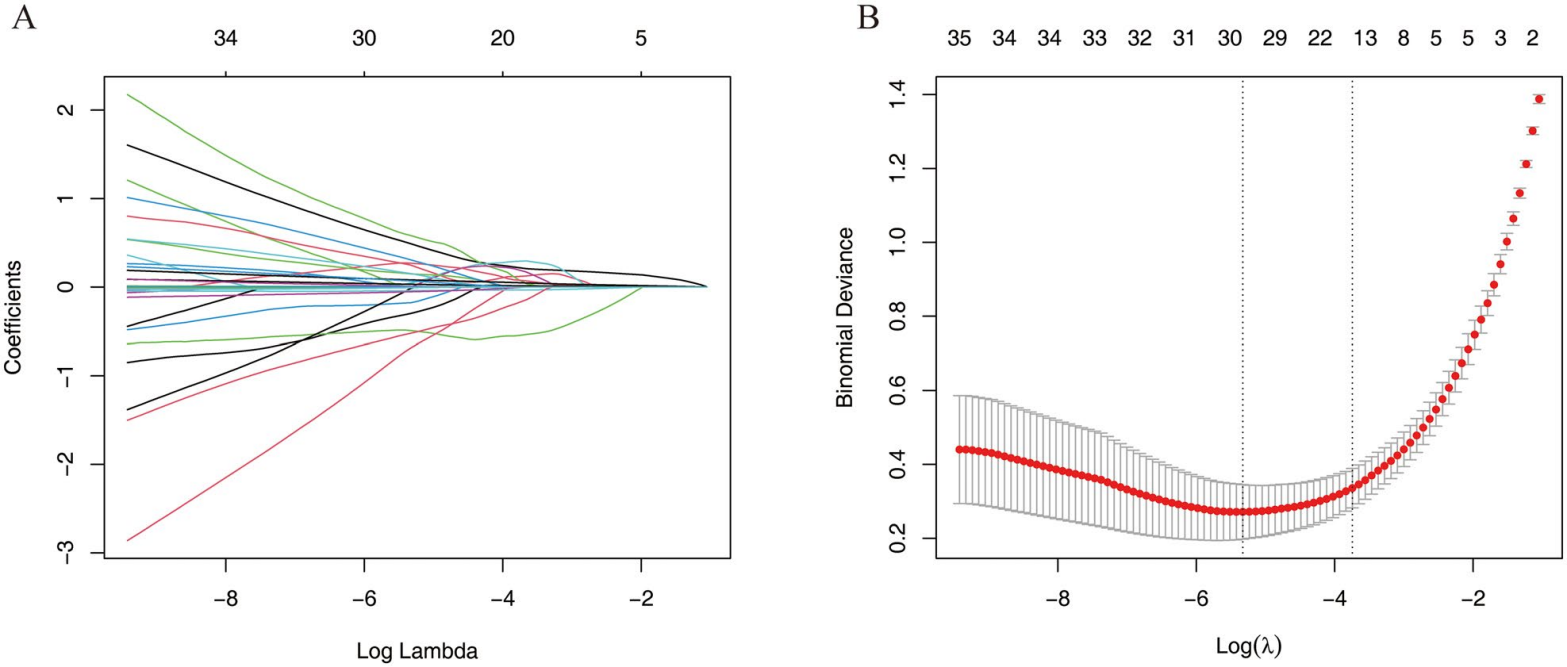
Screening predictors of ARDS using lasso regression. (A) LASSO coefficient profiles of the candidate predictors. (B) Selection of the optimal penalization coefficient.

**Figure 3.**
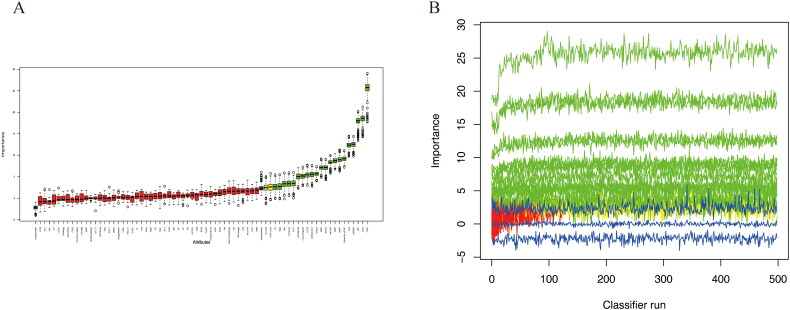
Selecting predictors of ARDS using random forest algorithm. (A) The boxplot shows the importance of each factor in the random forest algorithm. Boxplots in green, yellow, and blue were confirmed as important, tentative, and unimportant variables, respectively. (B) Decisions of rejecting or accepting features by random forest in 500 boruta function runs.

Lasso regression and random forest algorithm were used to select potential predictive factors. Factors associated with ARDS risk in AP patients in the literature were considered as candidate predictors for LASSO and RF algorithms. The Lasso coefficient profiles of features and the optimal penalization coefficient lambda (λ) are shown in Figure 2. LASSO regression analysis can minimize the potential collinearity and overfitting of variables. 15 variables were selected as optimal predictors by LASSO (Figure 2). The random forest algorithm is capable of dealing with a large number of variables, and tends to yield more accurate results. The result of random forest algorithm showed that 21 variables were related to ARDS (Figure 3).

**Figure 4.**
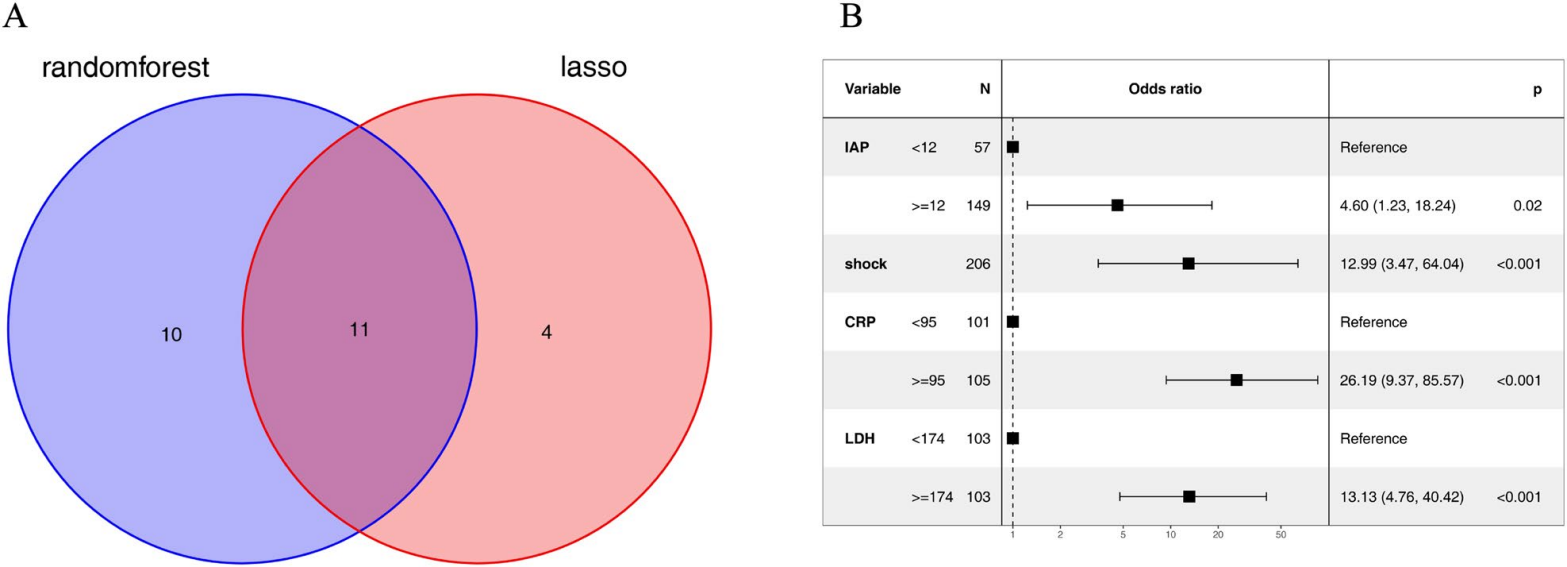
Multivariate logistic regression analysis. (A) The venn map used to illustrate the common predictors of ARDS. (B) Independent predictors assessed by multivariate logistic regression.

11 variables including Cr, Severity of AP, DBP, IAP, MODS, shock, neurological dysfunction, BISAP, PCT, CRP and LDH were screened as the common predictors of ARDS (Figure 4A).

### 3.3. Multivariate logistic regression

The stepwise method based on the common variables was conducted in the multivariable logistic regression analysis to identify the independent risk factors of ARDS. The results are presented in Figure 4B. It was shown that four variables including IAP (Odds Ratio (OR)=4.60, 95%CI:1.23-18.24, *p* = 0.02), shock (OR = 12.99, 95%CI:3.47-64.04, *p* < 0.001), CRP(OR= 26.19, 95%CI:9.37-85.57, *p* < 0.001) and LDH (OR = 13.13, 95%CI:4.76-40.42, *p* < 0.001) were correlated with ARDS (Figure 4B).

### 3.4. Development of the nomogram

**Figure 5.**
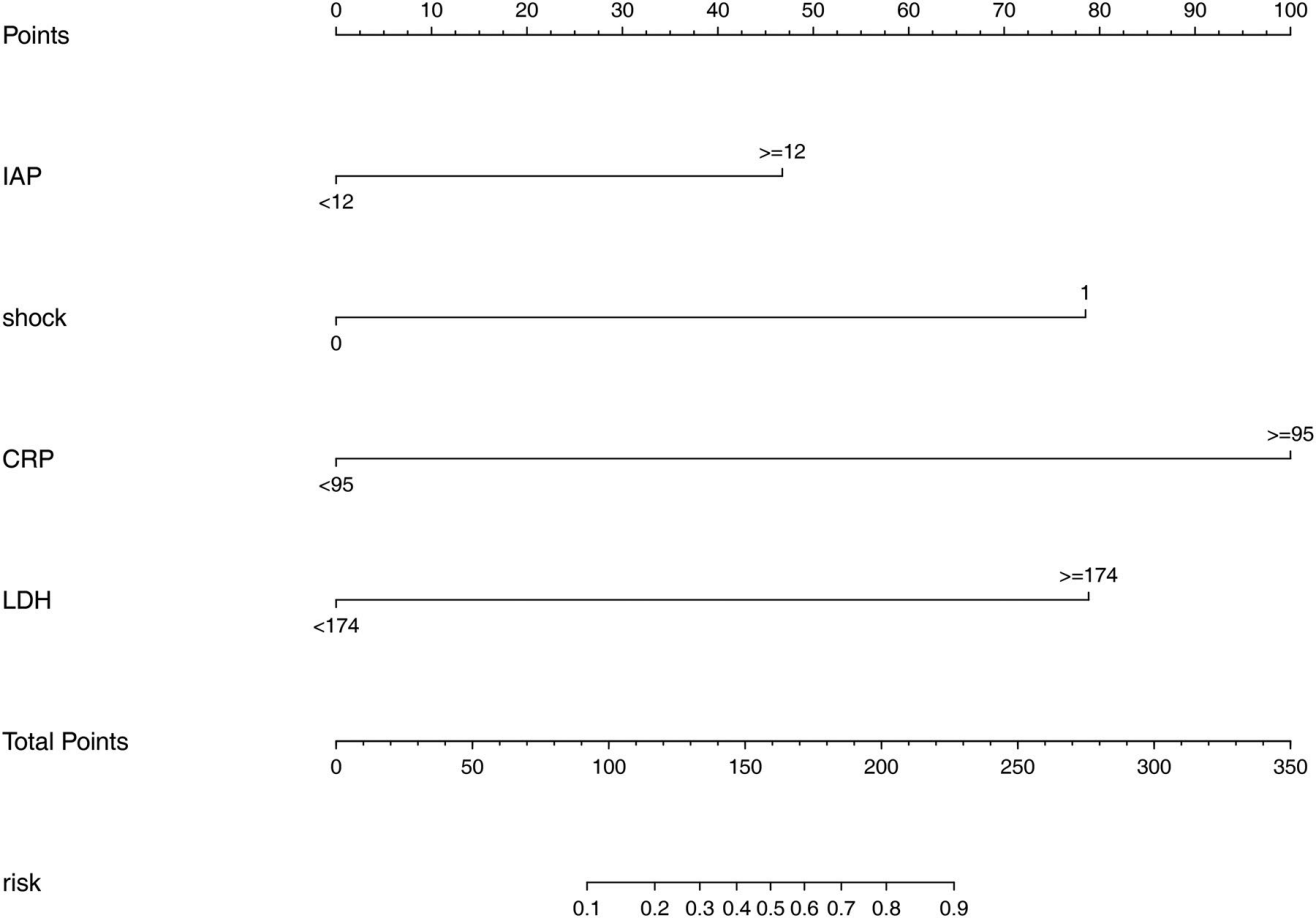
Nomogram predicting the probability of ARDS in AP patients complicated with AKI. Mark the individual’s value of each variable and obtain the points for each risk factor by drawing a vertical line from the variable axis to the “Points” axis. Then, cumulate the scores sum of all variables to obtain the total point. Finally, the probability of ARDS is obtained by drawing a vertical line from the “Total points” axis to “Risk” axis.

The four retained variables identified by multivariable logistic regression were then integrated to construct the predictive model (Figure 5). In the nomogram, points were assigned by drawing a vertical line from the variable axis to the “Points” axis. The total point is the cumulative sum of the points assigned to each variable. Finally, the probability of ARDS is obtained by drawing a vertical line from the “Total points” axis to “Risk” axis.

### 3.5. Validation, performance and clinical usefulness of the nomogram

**Figure 6.**
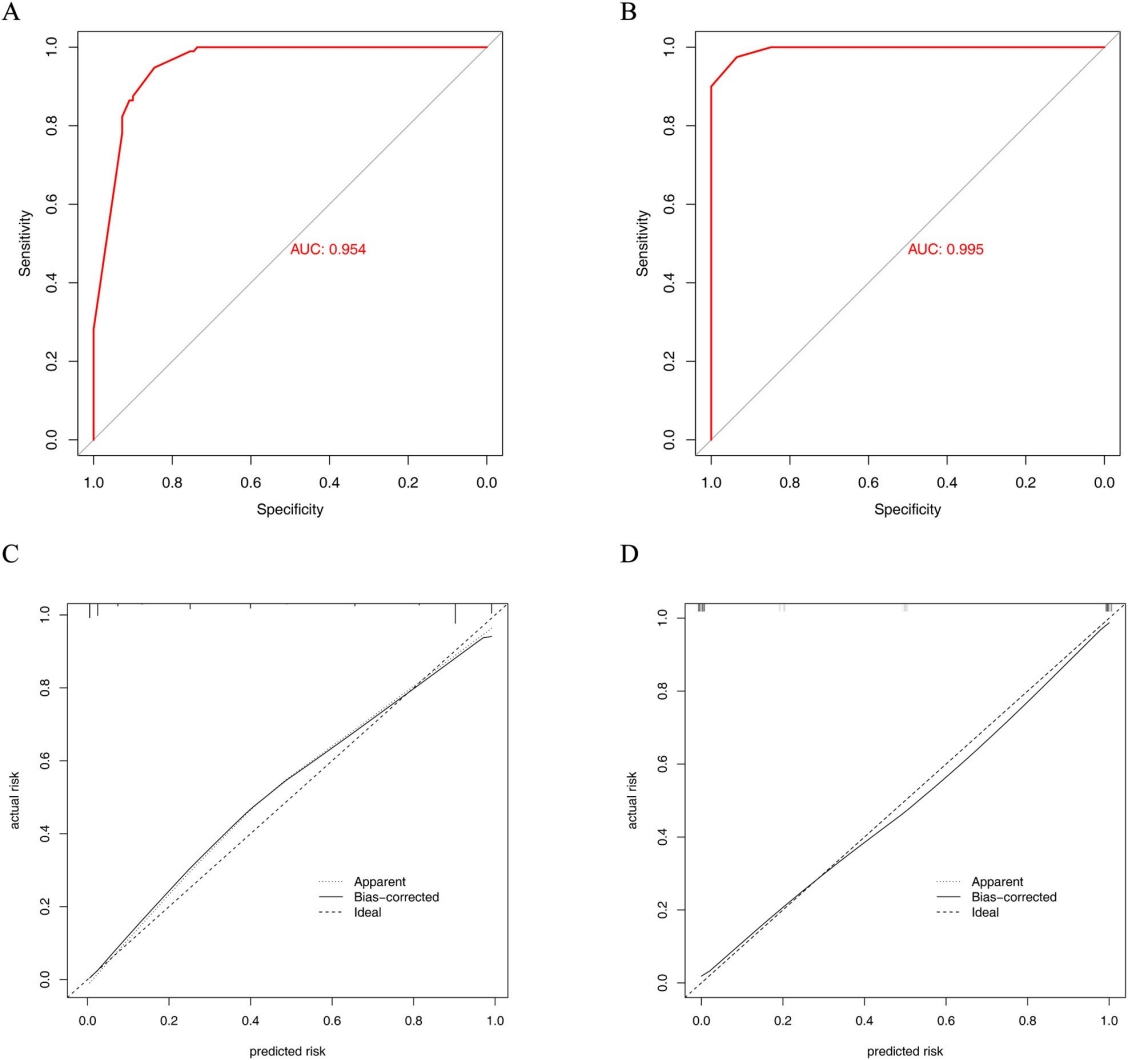
ROC curves and calibration curves of the nomogram. (A) ROC curve in the training cohort; (B) ROC curve in the validation cohort. (C) Calibration curve of the training cohort. (D) Calibration curve of the validation cohort.

Receiver operating characteristic (ROC) curves and calibration curves were used to evaluate the predictive performance of the nomogram. The discriminative ability of the nomogram was good with an AUC of 0.954 (95%CI: 0.928–0.979) in the training cohort and 0.995 (95%CI: 0.987–0.999) in the validation cohort (Figure 6(A and B)).

A calibration plot with bootstraps of 1000 resamples illustrated that the nomogram prediction was in accordance with the actual observation (Figure 6(C and D)).

**Figure 7.**
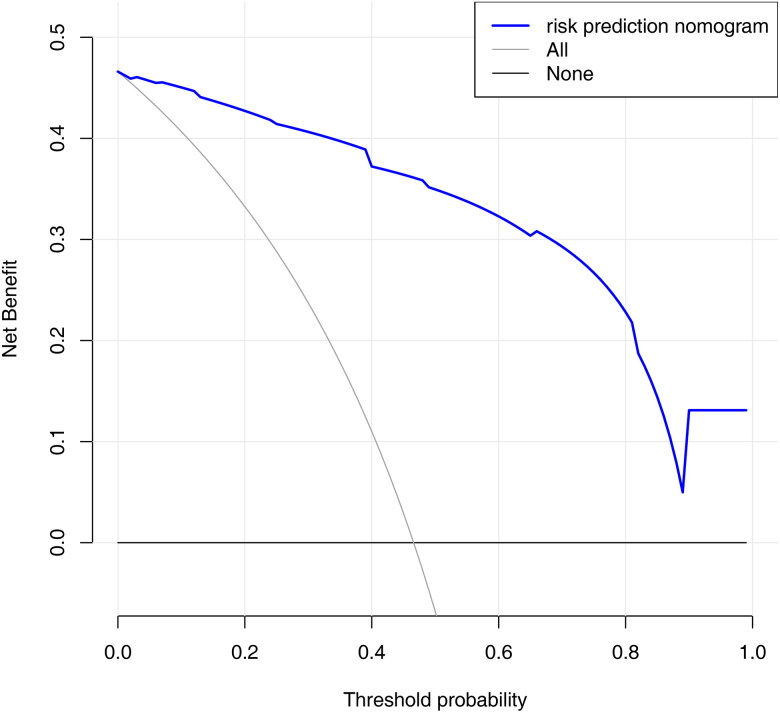
Decision curve analysis for the nomogram.

Decision curve analysis (DCA) was performed to evaluate the clinical usefulness of the predictive model. The results of DCA showed that using the nomogram provided a net benefit over “treat all” or “treat none” strategies (Figure 7). The range for the net benefit was 0.01-0.99.

## 4. Discussion

ARDS is one of the most common complications in AP patients [19], and is particularly problematic when patients complicated with AKI [3]. When ARDS and AKI overlap, the duration of hospitalization, resource utilization, and all‐cause mortality increase dramatically [20]. ARDS complicating AKI has imposed so heavy an burden on healthcare systems that it requires urgent attention. This study attempted to development an early predictive model for the occurrence of ARDS in AP patients complicated with AKI to help clinicians make rapid and accurate judgments on high-risk patients.

Machine learning algorithms are currently recognized as a powerful tool for clinical practice, which is capable of modeling information based on causal and/or statistical data, dealing with a large number of variables, and tend to construct more reliable diagnostic systems [21]. A nomogram based on clinically available data can early identify the high-risk population, assist clinicians in treatment management and prognosis estimation [22].

In our study, a nomogram consisiting of IAP, shock, CRP and LDH was developed to predict the risk of ARDS. To the best of our knowledge, this is the first visual nomogram to predict the risk of ARDS in AP patients complicated with AKI. This nomogram has reliable prediction performance, good calibration ability and can identify ARDS in a timely manner, which would be helpful for screening, clinical management, and prevention of disease progression.

ARDS is a type of rapidly progressive respiratory failure characterized by diffuse alveolar damage that is often accompanied by apoptosis of epithelial cells, alveolar and interstitial pulmonary edema, inflammatory cell infiltration, and pulmonary microthrombosis [23]. The pathophysiologic link between ARDS and AKI has been investigated, inflammatory cytokine storm, oxidative stress and dysregulation of ion/water transport channels play a crucial role in the development of ARDS complicating AKI [24]. Alternatively, with its attendant hypoxemia and mechanical ventilation-associated high PEEP, ARDS could increase renal vascular resistance, redistribute renal blood flow and activate neurohormonal pathways, leading to deteriorative renal hemodynamics [25]. Kidney-lung interactions contribute to an inflammatory loop that aggravates kidney injury and pulmonary vascular permeability [26–28].

Lung inflammation is persistent in the setting of AP [29], several studies have suggested the important role of cytokines/chemokines in the initiation and progression of ARDS after AKI [30]. Proinflammatory cytokines Interleukin (IL)-6, IL-8 and TNF are increased and directly injury the lung endothelium, leading to cardiogenic and non-cardiogenic pulmonary edema [31,32]. IL-6 and/or IL-8 were associated with prolonged mechanical ventilation and increased mortality in the setting of AKI [33,34]. Immune cell infiltration is the potential mediators of lung dysfunction during AKI [35], margination and trapping of neutrophils in the endothelium occurs in ALI and is considered central to its pathogenesis [4,32]. C-reactive protein (CRP), an acute phase protein produced by the liver, is elevated in critical conditions and represents characteristics of hyperinflammation [36]. CRP could be used as a biomarker to monitor the progression and improvement of diseases [37]. For many specialists, CRP is considered as the predictive factor for severity of acute pancreatitis [38]. Anti-inflammatory therapy targeting cytokines and modulation of oxidative stress has shown their potential as therapeutic targets [39]. The cornerstone of therapy for ARDS demonstrated to be a fluid conservative protocol. Maintaining appropriate fluid balance and ensuring adequate treatment of the underlying cause of ARDS are crucial. Renal replacement therapy(RRT) should be considered if diuretic therapy could not alleviate the exacerbation of fluid overload. Besides, RRT can help remove inflammatory mediators and maintain homeostasis, which is critical for improving the prognosis of critically ill patients. A recent study showed that CytoSorb coupled with hemodialysis provided a rescue therapy for shocked patients with systemic inflammatory response syndrome(SIRS) [40], suggesting that cytokine adsorption might be beneficial in ARDS patients complicated with AKI requiring RRT support.

LDH is an intracellular enzyme present in almost all organs, and is involved in the interconversion between pyruvate and lactate through an nicotinamide adenine dinucleotide (NADH)-dependent reaction [41]. The release of LDH was triggered by cytokine-mediated tissue damage in inflammatory disease [42]. Elevated LDH levels conferred a high sensitivity in early assessment and prediction of the worsening of AP [43,44]. The pancreatic inflammation lead to the capillary leak, intravascular depletion, third-spacing of fluid and even shock. Systemic haemodynamic instability very likely cause or aggravate injury of sensitive organs such as the kidneys and lungs without early intervention [45]. Consistent with previous studies, CRP, LDH and shock were included in the risk stratification model for ARDS in AP patients complicated with AKI.

Increased IAP has a multitude of effects on renal function through a series of mechanisms. Elevated IAP triggers decreased cardiac output and abdominal perfusion pressure, leading to renal hypoperfusion [46]. Additionally, compression of the renal parenchyma and renal venous system results in interstitial edema, elevation of intracapsular pressure, and declined glomerular filtration fraction [47]. Acute tubular necrosis and the activation of renin-angiotensin-aldosterone system (RAAS) may also be involved in AKI.

Intra-abdominal hypertension (IAH) is associated with increased intra-thoracic pressure, decreased respiratory sysytem compliance and functional residual capacity (FRC), leading to basilar atelectasis, airway closure, deterioration of respiratory mechanics and reduced gas exchange [48]. Patients with IAH are more likely to develop ARDS and even need mechanical ventilation [49]. Elevated IAP has adverse effects on respiratory system through the kidney-lung interaction. Therefore, monitoring IAP has potential significance for the clinical management of AP patients.

To the best of our knowledge, it is the first model to predict ARDS in AP patients complicated with AKI. The strength of our study is that the predictive variables are all taken from routine clinical tests and the nomogram is easier for clinicians to use than the APACHE-II, Ranson, CTSI and BISAP scoring systems. Secondly, Lasso regression analysis and machine learning algorithms were both applied to select characteristic factors. The dimension reduction in penalized regression methods and optimization process of datasets with a large number of variables can minimize the potential collinearity and overfitting of variables, thus improving the prediction accuracy.

However, this study was subject to some limitations. Firstly, this is a single-center retrospective study, which may have some selection bias. A multicenter prospective study must further verify the predictive effect of the nomogram. Secondly, some novel biomarkers (such as angiopoietin-2, IL-6, IL-8 and IL-10) are not included in our predictive nomogram, which may limit the predictive performance. But these indicators were not part of the routine clinical practice.

## 5. Conclusions

Based on a simplified model consisting of four variables, a nomogram was drawn to predict the risk of ARDS in AP patients complicated with AKI. The nomogram offers support for developing timely prevention and intervention strategies for AP patients complicated with AKI.

## Data Availability

The datasets used in the current study are available from the corresponding author if required.

## References

[1] Ge P, Luo Y, Okoye CS, et al. Intestinal barrier damage, systemic inflammatory response syndrome, and acute lung injury: a troublesome trio for acute pancreatitis. Biomed Pharmacother. 2020;132:1. doi: 10.1016/j.biopha.2020.110770. 33011613

[2] Mayerhöfer T, Perschinka F, Joannidis M. [Acute kidney injury and COVID-19: lung-kidney crosstalk during severe inflammation]. Med Klin Intensivmed Notfmed. 2022;117(5):342–16. doi: 10.1007/s00063-022-00919-3. 35476144 PMC9044389

[3] Jaber S, Garnier M, Asehnoune K, et al. Guidelines for the management of patients with severe acute pancreatitis, 2021. Anaesth Crit Care Pain Med. 2022;41(3):101060. doi: 10.1016/j.accpm.2022.101060. 35636304

[4] Nakazawa D, Marschner JA, Platen L, et al. Extracellular traps in kidney disease. Kidney Int. 2018;94(6):1087–1098. doi: 10.1016/j.kint.2018.08.035. 30466565

[5] Griffin BR, Liu KD, Teixeira JP. Critical care nephrology: core curriculum 2020. Am J Kidney Dis. 2020;75(3):435–452. doi: 10.1053/j.ajkd.2019.10.010. 31982214 PMC7333544

[6] Park BD, Faubel S. Acute kidney injury and acute respiratory distress syndrome. Crit Care Clin. 2021;37(4):835–849. doi: 10.1016/j.ccc.2021.05.007. 34548136 PMC8157315

[7] Uddin S, Khan A, Hossain ME, et al. Comparing different supervised machine learning algorithms for disease prediction. BMC Med Inform Decis Mak. 2019;19(1):281. doi: 10.1186/s12911-019-1004-8. 31864346 PMC6925840

[8] Chen Z, Chen C, Chen F, et al. Bioinformatics analysis of potential pathogenesis and risk genes of immunoinflammation-promoted renal injury in severe COVID-19. Front Immunol. 2022;13:950076. doi: 10.3389/fimmu.2022.950076. 36052061 PMC9424635

[9] Leppäniemi A, Tolonen M, Tarasconi A, et al. 2019 WSES guidelines for the management of severe acute pancreatitis. World J Emerg Surg. 2019;14(1):27. doi: 10.1186/s13017-019-0247-0. 31210778 PMC6567462

[10] Kryvoruchko IA, Kopchak VM, Usenko O[, et al. Classification of an acute pancreatitis: revision by international consensus in 2012 of classification, adopted in atlanta]. Klin Khir. 2014 Sep;(9):19–24. 25509427

[11] Khwaja A. KDIGO clinical practice guidelines for acute kidney injury. Nephron Clin Pract. 2012;120(4):c179–184. doi: 10.1159/000339789. 22890468

[12] Force ADT, Ranieri VM, Rubenfeld GD, et al. Acute respiratory distress syndrome: the Berlin definition. Jama. 2012;307(23):2526–2533. 22797452 10.1001/jama.2012.5669

[13] Matthay MA, Arabi Y. A New Global Definition of Acute Respiratory Distress Syndrome. 2023. doi: 10.1164/ajrccm-conference.2023.207.1_MeetingAbstracts.A6229. PMC10870872 37487152

[14] Kirkpatrick AW, Roberts DJ, De Waele J, et al. Intra-abdominal hypertension and the abdominal compartment syndrome: updated consensus definitions and clinical practice guidelines from the world society of the abdominal compartment syndrome. Intensive Care Med. 2013;39(7):1190–1206. doi: 10.1007/s00134-013-2906-z. 23673399 PMC3680657

[15] Shankar-Hari M, Phillips GS, Levy ML, et al. Developing a new definition and assessing new clinical criteria for septic shock: for the third international consensus definitions for sepsis and septic shock (sepsis-3). Jama. 2016;315(8):775–787. doi: 10.1001/jama.2016.0289. 26903336 PMC4910392

[16] Cho JH, Kim TN, Chung HH, et al. Comparison of scoring systems in predicting the severity of acute pancreatitis. World J Gastroenterol. 2015;21(8):2387–2394. doi: 10.3748/wjg.v21.i8.2387. 25741146 PMC4342915

[17] Kokla M, Virtanen J, Kolehmainen M, et al. Random Forest-based imputation outperforms other methods for imputing LC-MS metabolomics data: a comparative study. BMC Bioinformatics. 2019;20(1):492. doi: 10.1186/s12859-019-3110-0. 31601178 PMC6788053

[18] Zhang Z. Multiple imputation with multivariate imputation by chained equation (MICE) package. Annals of Translational Medicine. 2016;4(2):30. 26889483 10.3978/j.issn.2305-5839.2015.12.63 PMC4731595

[19] Zhou MT, Chen CS, Chen BC, et al. Acute lung injury and ARDS in acute pancreatitis: mechanisms and potential intervention. World J Gastroenterol. 2010;16(17):2094–2099. doi: 10.3748/wjg.v16.i17.2094. 20440849 PMC2864834

[20] Basu RK, Wheeler DS. Kidney-lung cross-talk and acute kidney injury. Pediatr Nephrol. 2013;28(12):2239–2248. doi: 10.1007/s00467-012-2386-3. 23334385 PMC5764184

[21] Handelman GS, Kok HK, Chandra RV, et al. eDoctor: machine learning and the future of medicine. J Intern Med. 2018;284(6):603–619. doi: 10.1111/joim.12822. 30102808

[22] Wang R, Dai W, Gong J, et al. Development of a novel combined nomogram model integrating deep learning-pathomics, radiomics and immunoscore to predict postoperative outcome of colorectal cancer lung metastasis patients. J Hematol Oncol. 2022;15(1):11. 10.1186/s13045-022-01225-3 PMC8785554 35073937

[23] Meyer NJ, Gattinoni L, Calfee CS. Acute respiratory distress syndrome. Lancet. 2021;398(10300):622–637. doi: 10.1016/S0140-6736(21)00439-6. 34217425 PMC8248927

[24] Herrlich A. Interorgan crosstalk mechanisms in disease: the case of acute kidney injury-induced remote lung injury. FEBS Lett. 2022;596(5):620–637. doi: 10.1002/1873-3468.14262. 34932216

[25] Vashisht R, Duggal A. Acute kidney injury in acute respiratory distress syndrome: why ventilator settings matter. Ann Transl Med. 2020;8(9):573–573. doi: 10.21037/atm-20-2163. 32566600 PMC7290557

[26] Wang Z, Pu Q, Huang C, et al. Crosstalk between lung and extrapulmonary organs in infection and inflammation. Adv Exp Med Biol. 2021;1303:333–350. doi: 10.1007/978-3-030-63046-1_18. 33788201

[27] Husain-Syed F, Rosner MH, Ronco C. Distant organ dysfunction in acute kidney injury. Acta Physiol (Oxf). 2020;228(2):e13357. doi: 10.1111/apha.13357. 31379123

[28] Alge J, Dolan K, Angelo J, et al. Two to tango: kidney-Lung interaction in acute kidney injury and acute respiratory distress syndrome. Front Pediatr. 2021;9:744110. doi: 10.3389/fped.2021.744110. 34733809 PMC8559585

[29] Samanta J, Singh S, Arora S, et al. Cytokine profile in prediction of acute lung injury in patients with acute pancreatitis. Pancreatology. 2018;18(8):878–884. doi: 10.1016/j.pan.2018.10.006. 30361069

[30] Ahuja N, Andres-Hernando A, Altmann C, et al. Circulating IL-6 mediates lung injury via CXCL1 production after acute kidney injury in mice. Am J Physiol Renal Physiol. 2012;303(6):F864–872. doi: 10.1152/ajprenal.00025.2012. 22791336 PMC3468527

[31] Altmann C, Andres-Hernando A, McMahan RH, et al. Macrophages mediate lung inflammation in a mouse model of ischemic acute kidney injury. Am J Physiol Renal Physiol. 2012;302(4):F421–432. doi: 10.1152/ajprenal.00559.2010. 22114207 PMC3289418

[32] Faubel S, Edelstein CL. Mechanisms and mediators of lung injury after acute kidney injury. Nat Rev Nephrol. 2016;12(1):48–60. doi: 10.1038/nrneph.2015.158. 26434402

[33] Liu KD, Altmann C, Smits G, et al. Serum interleukin-6 and interleukin-8 are early biomarkers of acute kidney injury and predict prolonged mechanical ventilation in children undergoing cardiac surgery: a case-control study. Crit Care. 2009;13(4):R104. doi: 10.1186/cc7940. 19570208 PMC2750143

[34] Tomasi A, Song X, Gajic O, et al. Kidney and lung crosstalk during critical illness: large-scale cohort study. 2023;36(4):1037–1046. 10.1007/s40620-022-01558-9 36692665

[35] Jing W, Qin F, Guo X, et al. G-CSF mediates lung injury in mice with adenine-induced acute kidney injury. Int Immunopharmacol. 2018;63:1–8. doi: 10.1016/j.intimp.2018.07.032. 30056257

[36] Mehta P, McAuley DF, Brown M, et al. COVID-19: consider cytokine storm syndromes and immunosuppression. Lancet. 2020;395(10229):1033–1034. doi: 10.1016/S0140-6736(20)30628-0. 32192578 PMC7270045

[37] Ponti G, Maccaferri M, Ruini C, et al. Biomarkers associated with COVID-19 disease progression. Crit Rev Clin Lab Sci. 2020;57(6):389–399. doi: 10.1080/10408363.2020.1770685. 32503382 PMC7284147

[38] Sternby H, Hartman H, Johansen D, et al. IL-6 and CRP are superior in early differentiation between mild and non-mild acute pancreatitis. Pancreatology. 2017;17(4):550–554. doi: 10.1016/j.pan.2017.05.392. 28610827

[39] Tai H, Jiang XL, Song N, et al. Tanshinone IIA combined with cyclosporine a alleviates lung apoptosis induced by renal Ischemia-Reperfusion in obese rats. Front Med (Lausanne). 2021;8:617393. doi: 10.3389/fmed.2021.617393. 34012969 PMC8126627

[40] Persic V, Jerman A. Effect of CytoSorb coupled with hemodialysis on interleukin-6 and hemodynamic parameters in patients with systemic inflammatory response syndrome: a retrospective cohort study. J Clin Med. 2022;11(24):7500. 10.3390/jcm11247500 PMC9788171 36556116

[41] Hsu PP, Sabatini DM. Cancer cell metabolism: warburg and beyond. Cell. 2008;134(5):703–707. doi: 10.1016/j.cell.2008.08.021. 18775299

[42] Malik P, Patel U, Mehta D, et al. Biomarkers and outcomes of COVID-19 hospitalisations: systematic review and meta-analysis. BMJ Evid Based Med. 2021;26(3):107–108. doi: 10.1136/bmjebm-2020-111536. PMC7493072 32934000

[43] Asim Riaz HM, Islam Z, Rasheed L, et al. The evaluation of inflammatory biomarkers in predicting progression of acute pancreatitis to pancreatic necrosis. A Diagnostic Test Accuracy Review. 2022;11(1):27. 10.3390/healthcare11010027 PMC9818910 36611486

[44] Komolafe O, Pereira SP, Davidson BR, et al. Serum C-reactive protein, procalcitonin, and lactate dehydrogenase for the diagnosis of pancreatic necrosis. Cochrane Database Syst Rev. 2017;4(4):Cd012645. doi: 10.1002/14651858.CD012645. 28431197 PMC6478063

[45] Gad MM, Simons-Linares CR. Is aggressive intravenous fluid resuscitation beneficial in acute pancreatitis? A meta-analysis of randomized control trials and cohort studies. World J Gastroenterol. 2020;26(10):1098–1106. doi: 10.3748/wjg.v26.i10.1098. 32206000 PMC7081000

[46] Copur S, Berkkan M, Hasbal NB, et al. Abdominal compartment syndrome: an often overlooked cause of acute kidney injury. J Nephrol. 2022;35(6):1595–1603. doi: 10.1007/s40620-022-01314-z. 35380354

[47] Mohmand H, Goldfarb S. Renal dysfunction associated with intra-abdominal hypertension and the abdominal compartment syndrome. J Am Soc Nephrol. 2011;22(4):615–621. doi: 10.1681/ASN.2010121222. 21310818

[48] Pereira BM. Abdominal compartment syndrome and intra-abdominal hypertension. Curr Opin Crit Care. 2019;25(6):688–696. doi: 10.1097/MCC.0000000000000665. 31524716

[49] Regli A, Reintam Blaser A, De Keulenaer B, et al. Intra-abdominal hypertension and hypoxic respiratory failure together predict adverse outcome – a sub-analysis of a prospective cohort. J Crit Care. 2021;64:165–172. doi: 10.1016/j.jcrc.2021.04.009. 33906106

